# New Developments in Peritoneal Fibroblast Biology: Implications for Inflammation and Fibrosis in Peritoneal Dialysis

**DOI:** 10.1155/2015/134708

**Published:** 2015-10-01

**Authors:** Janusz Witowski, Edyta Kawka, Andras Rudolf, Achim Jörres

**Affiliations:** ^1^Department of Pathophysiology, Poznan University of Medical Sciences, Medical Biology Centre, Rokietnicka 8, 60-806 Poznan, Poland; ^2^Department of Nephrology and Medical Intensive Care, Charité-Universitätsmedizin Berlin, Campus Virchow-Klinikum, Augustenburger Platz 1, 13353 Berlin, Germany

## Abstract

Uraemia and long-term peritoneal dialysis (PD) can lead to fibrotic thickening of the peritoneal membrane, which may limit its dialytic function. Peritoneal fibrosis is associated with the appearance of myofibroblasts and expansion of extracellular matrix. The extent of contribution of resident peritoneal fibroblasts to these changes is a matter of debate. Recent studies point to a significant heterogeneity and complexity of the peritoneal fibroblast population. Here, we review recent developments in peritoneal fibroblast biology and summarize the current knowledge on the involvement of peritoneal fibroblasts in peritoneal inflammation and fibrosis.

## 1. Introduction

Fibroblasts are the commonest connective tissue cells and the main source of extracellular matrix. Until recently, fibroblasts have been viewed as cells providing only structural framework for tissues. Now it is clear that fibroblasts are at the center of tissue homeostasis and serve specialized functions in different organs. Impressive versatility of fibroblasts is reflected by differences in gene expression patterns according to anatomic location [1]. Moreover, even the same tissue can be populated with several fibroblast subsets with distinct functions [2]. The phenotype of fibroblasts may change further during wound healing or fibrosis, when cells become activated and termed “myofibroblasts.” When analyzing fibroblasts, it is therefore essential to take the exact physiological and clinical context into account. Here, we review new developments in our understanding of the role of fibroblasts in the peritoneum, especially their involvement in peritoneal dialysis- (PD-) associated fibrosis.

## 2. Fibroblast Identity and Phenotype

Normal resident tissue fibroblasts are identified by their spindle-shape appearance and location within the connective tissue. They may also express fibroblast-specific protein-1 (FSP-1), but not molecular markers for other cell types. In response to tissue injury and stimulation with growth factors (e.g., TGF-*β*), fibroblasts can adopt an activated phenotype resembling that of smooth muscle cells and characterized by the expression of smooth muscle actin (*α*-SMA). The resulting myofibroblasts have increased contractile capacity and show increased proliferation and motility. They produce more extracellular matrix (ECM) components and more regulators of the ECM turnover. The origin of myofibroblasts in different tissues (including the peritoneum) is a matter of intensive research and some controversy. Classically, myofibroblasts were thought to derive from resident tissue fibroblasts. However, there are other potential precursors of myofibroblasts, including epithelial, mesothelial, and endothelial cells, as well as bone marrow-derived fibrocytes. Introduction of new fibroblast biomarkers and great advances in lineage tracing techniques allowed us to better define the contribution of these cells to adverse tissue remodeling.

**Figure 1:**
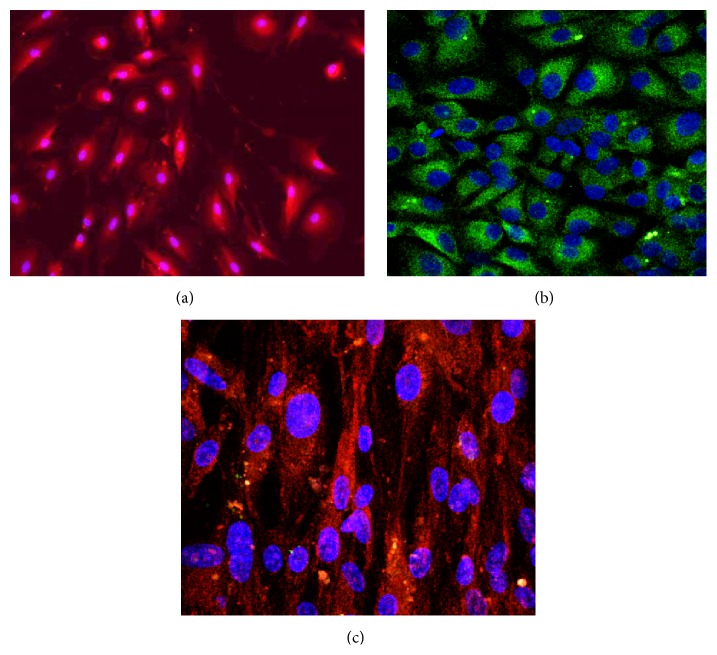
Expression of FSP-1 by human peritoneal fibroblasts and mesothelial cells. Human peritoneal mesothelial cells and peritoneal resident fibroblasts were isolated from apparently normal omentum by enzymatic digestion, as described [5]. Populations of mesothelial cells and fibroblasts were immunostained for FSP-1 (red) and cytokeratin (green). Nuclei were counterstained with DAPI (blue). Magnification 200x. (a) Human peritoneal fibroblasts express FSP-1 but not cytokeratin. (b) Human peritoneal mesothelial cells express cytokeratin but not FSP-1. (c) Stimulation of mesothelial cells with TGF-*β*1 (1 ng/mL; 72 hours) leads to mesenchymal transition that is associated with loss of cytokeratin expression and de novo FSP-1 expression.

The analysis of peritoneal membrane biopsies from patients treated with PD has clearly demonstrated that the thickness of the submesothelial compact zone, a layer of mature fibrous tissue containing collagen and elastin fibers, progressively increases with duration of PD [3]. This is particularly evident in the parietal peritoneum [4]. Thickening of the peritoneum reflects the expansion of ECM, which is deposited primarily by myofibroblasts. To determine the origin of myofibroblasts, it is necessary to identify first resident fibroblasts and mesothelial cells. This has not always been an easy task, either in vivo or in culture (see [5] for a review). Mesothelial cells display typically an epithelial-like appearance and form a monolayer covering the peritoneal surface, while fibroblasts are fusiform cells embedded in the submesothelial interstitium. In the course of mesenchymal transition, however, mesothelial cells acquire a fibroblast-like phenotype, become motile, and invade the underlying stroma. Moreover, both fibroblasts and mesothelial arise from the mesoderm and can share rather than differ in certain biomarkers, for example, vimentin. Therefore, the identification of FSP-1, a calcium-binding protein of the S100 family, as providing better specificity for fibroblasts [6, 7] was viewed as a great step forward in tracking fibroblasts. However, the validity of FSP-1 as the specific fibroblast marker has been questioned. It turned out that FSP-1 might be absent in a proportion of normal interstitial fibroblasts and some FSP-1-positive cells in diseased tissues might be, in fact, mononuclear or endothelial cells [8]. Despite these uncertainties, FSP-1 in combination with other biomarkers is still used in studies on the origin of peritoneal myofibroblasts [9, 10]. In cell culture, antibodies against FSP-1 can help purify peritoneal fibroblasts from contaminating mesothelial cells [11] (Figure 1).

## 3. Fibroblast Subsets

In addition to FSP-1, further fibroblast subsets can be identified using other criteria. Thy-1 (CD90), a glycophosphatidylinositol-linked outer cell membrane protein, is expressed by many cell types, but it can separate fibroblasts into Thy-1^+^ and Thy-1^−^ subpopulations with different phenotypic and functional features [12–15]. The significance of this trait may differ according to the origin of fibroblasts. Normal lung fibroblasts, both in mice and in humans, are predominantly Thy-1-positive and their presence appears to limit pulmonary fibrosis [15–18]. It has been demonstrated that the absence of Thy-1 on lung fibroblasts is associated with their myofibroblastic phenotype and enhanced proliferative response to fibrogenic stimuli. Mice deficient in Thy-1 show exaggerated fibrosis and myofibroblastic differentiation after bleomycin-induced pulmonary injury. In humans with idiopathic pulmonary fibrosis, no Thy-1 staining was seen in fibroblastic foci. These results suggest that the loss of lung fibroblast Thy-1 expression after injury promotes enhanced fibrogenesis. In contrast to lung fibroblasts, however, the myofibroblastic conversion of orbital and myometrial fibroblasts in response to TGF-*β* appears to be favored by the presence rather than the absence of Thy-1 [12].

Little is known about the role of Thy-1 in peritoneal cells. A small population of Thy-1^+^ (CD90^+^) mesothelial-like cells has recently been detected in ascites drained from patients with gastrointestinal cancers [19]. These cells were defined as mesenchymal stem cells and showed a distinct myofibroblastic phenotype after stimulation with TGF-*β*. We have examined Thy-1 expression patterns in apparently normal human peritoneal fibroblasts (HPFB) in culture. Indeed, it appears that both Thy-1^+^ and Thy-1^−^ subsets of HPFB exist in the peritoneum and differ in morphology and the ability to acquire a myofibroblastic phenotype (Kawka E. et al., personal observations).

## 4. Resident Peritoneal Fibroblasts

Fibroblasts of the normal peritoneum are scattered in the submesothelial connective tissue. Electron microscopy shows large multipolar cells embedded between collagen and elastic fibers [20]. These cells express neither myofibroblastic nor mesothelial markers [21]. Also, the expression of FSP-1 is not evident [9, 22] (Kawka E. et al., personal observations), which adds to the reservations about FSP-1 marking resident fibroblasts in the normal peritoneum [8]. On the other hand, the cells may bear platelet-derived growth factor receptor-*β* (PDGFR*β*, CD140b) [23], which is sometimes used as a marker of resident fibroblasts. However, PDGFR*β* is also expressed by pericytes and the exact relationship between pericytes and perivascular fibroblasts is not clear [24].

The cells identified as submesothelial fibroblasts occasionally express hematopoietic cell surface marker CD34 [21], which may indicate that they are derived from blood-borne fibrocytes. But they do not usually express other fibrocyte markers (CD45, CD11b, and MHC class II), suggesting that they are rather primal mesenchymal cells [25] residing in the peritoneum.

The thickness of the submesothelial compact zone in uremic patients is already increased before the commencement of dialysis, pointing to a detrimental impact of uraemia itself [3, 26]. Nevertheless, the phenotype of resident fibroblasts and their biomarker expression patterns do not seem to be altered significantly [21]. PD exposure leads to further thickening of the compact zone and distinct changes in peritoneal fibroblasts. The presence of FSP-1 expression becomes evident [9, 10], although it is not clear whether this comes from resident fibroblasts or other cell types transitioning into fibroblasts (see below). Soon after the initiation of PD many fibroblasts acquire a myofibroblastic phenotype as evidenced by *α*-SMA expression [21, 27, 28]. They often form clusters and localize immediately beneath the mesothelial surface. They may also lose CD34 expression and show cytokeratin and E-cadherin expression instead [21]. The significance of CD34 loss is unclear, although it has been observed in fibrotic lesions in other tissues [29]. On the other hand, the expression of cytokeratin and E-cadherin by myofibroblasts is viewed as an indication of their origin from mesothelial cells [30].

## 5. Epithelial-to-Mesenchymal Transition as a Source of Peritoneal Fibroblasts

The concept of peritoneal myofibroblasts arising from the mesothelium through epithelial-to-mesenchymal transition (EMT) received a lot of attention over the past decade (see [25, 31–33] for excellent reviews). It appears that in the course of PD mesothelial cells upregulate transcription factors of the snail, ZEB, and Twist families, which control molecular reprogramming of EMT [34]. The whole process can be induced by a number of growth factors, including transforming growth factor-*β* (TGF-*β*), which has been identified as a key mediator of mesothelial EMT both in vitro [35] (Figure 1) and in vivo [36]. TGF-*β* exerts its effects by engaging various members of the family of mitogen-activated protein kinases (MAPKs), including TGF-*β*-activated kinase-1 (TAK-1) [37], p38 [38], and JNK kinase [39]. Inhibition of MAPK phosphorylation with glucocorticoids can block TGF-*β*-induced EMT of mesothelial cells in culture [40]. In addition to MAPKs, the involvement of the nuclear transcription factor-*κ*B (NF-*κ*B) in mesothelial cell transition has been postulated [41]. In vivo, adenovirus-mediated overexpression of TGF-*β* in the rat peritoneum results in the upregulation of several genes involved in EMT (snail, collagen 1, and *α*-SMA) [36]. In this setting the cytokeratin/*α*-SMA double-positive cells appear first in the mesothelial monolayer and later in the reorganized submesothelial matrix [36]. These effects are mediated by both SMAD3-dependent and SMAD3-independent mechanisms [42]. The fibroblast-like phenotype of mesothelial cells isolated from PD patients or treated in vitro with TGF-*β* could be reversed by bone morphogenic protein-7 (BMP-7) [43] or by TAK-1 inhibitors [37]. Of particular interest in the context of PD is the observation that exposure of mesothelial cells in vitro to high glucose can induce Twist, a key EMT-controlling transcription factor [44], and increase the expression of *α*-SMA [45]. These effects could be related to upregulation of TGF-*β* by high glucose [46], but they can also be attributed to decreased expression of BMP-7 [45] or heme oxygenase-1 (HO-1) [47]. Indeed, experimental upregulation of BMP-7 or HO-1 could partly reduce high glucose-induced EMT of mesothelial cells. In an animal model of PD, adenovirus-mediated transfection of BMP-7 was found to inhibit EMT in mesothelial cells and decrease subsequent peritoneal thickening [45].

What still remains unclear is the mechanism by which PD exposure initiates EMT. The analysis of peritoneal membrane biopsies revealed that the loss of mesothelial cells from the peritoneal surface and the appearance of submesothelial cytokeratin staining occur relatively often and early during PD [49]. The observation that the number of fibroblast-like mesothelial cells isolated from spent dialysate effluent increases with the duration of therapy [30] points to the role of cumulative exposure to PD fluids and/or occasional episodes of peritonitis. In this respect, it has been demonstrated that key proinflammatory cytokines IL-1*β* and TNF*α* induce increased peritoneal TGF-*β* expression in animal models [50]. Moreover, there exists data to suggest a link between EMT and biocompatibility of PD solutions. It has been observed that PD fluids with high concentration of glucose degradation products (GDP) can induce EMT in mesothelial cells both during short-term direct exposure in culture and—to lesser extent—after chronic PD exposure in vivo [51, 52]. This finding is in line with earlier observations of EMT in the peritoneal membrane of rats treated with chronic intraperitoneal administration of GDP [53].

Loureiro and colleagues have assessed the exact contribution of various precursors to the pool of peritoneal fibroblasts that accumulate in the peritoneum of mice during chronic PD [9]. They found no FSP-1^+^ cells in the normal peritoneum. However, such cells did appear after exposure to PD fluids and could be further characterized by dual-immunolabeling using an anti-FSP-1 antibody in conjunction with antibodies against cytokeratin, CD45, or CD31. Approximately 37% FSP-1^+^ cells were identified as derived from mesothelial cells (FSP-1^+^/cytokeratin^+^), 34% from fibrocytes (FSP-1^+^/CD45^+^), and 5% from endothelial cells (FSP-1^+^/CD31^+^). The remaining 24% cells stained singly for FSP-1 and their origin was not defined. Interestingly, by using three-color immunofluorescence it has been estimated that approximately 50% of FSP-1^+^/cytokeratin^+^ cells coexpressed *α*-SMA, which pointed to their myofibroblastic phenotype. Importantly, the administration of TGF-*β*-blocking peptides significantly reduced the extent of PD fluid-induced peritoneal fibrosis and the number of FSP-1^+^ cells, especially of the FSP-1^+^/cytokeratin^+^ subpopulation. More recently, the same group have demonstrated that TGF-*β* can be also involved in mesenchymal transition of mesothelial cells induced by endothelin-1 [54]. These data support the concept that peritoneal fibrosis in PD is largely related to TGF-*β*-driven conversion of mesothelial cells into myofibroblasts.

The potential of mesothelial cells to undergo EMT and to contribute to other cell lineages has been well documented during development. In particular, it has been demonstrated that the pleural mesothelium is a source of peribronchiolar fibroblasts in the foetal lung and that the process of mesothelial cell migration into the lung parenchyma is controlled by the hedgehog signaling pathway [55]. Moreover, it appears that such a process may also occur in adult tissues. In this respect, mesothelial cells covering the liver were found to differentiate into hepatic myofibroblasts during liver injury and fibrosis [56]. Similarly, EMT in pleural mesothelial cells was found to be contributing to idiopathic pulmonary fibrosis [57]. Interestingly, several of the above studies employed the Cre recombinase technology to trace the fate of mesothelial cells that expressed the Wilms tumor-1 (Wt-1) transcription factor as a biomarker. Genetic mapping of Wt-1^+^ cells has also been used recently by Chen and colleagues to identify the cellular origin of myofibroblasts during peritoneal fibrosis [58]. The results of their study posed a challenge to the relevance of mesothelial cells as a source of peritoneal myofibroblasts. In contrast to earlier studies, they have observed that these were submesothelial resident fibroblasts rather than mesothelial cells that gave rise to collagen-producing myofibroblasts after injury induced by sodium hypochlorite (and to lesser extent by PD solutions or by adenovirus-mediated TGF-*β*1 overexpression). Resident fibroblasts were defined as cytokeratin^−^, vimentin^+^, and PDGF*β* ^+^ cells located beneath mesothelial cell basement membrane. Interestingly, the use of PDGFR inhibitor after injury significantly attenuated the accumulation of *α*SMA^+^ myofibroblasts and peritoneal fibrosis. Obviously, the results of this study will need to be independently confirmed, given the limitations of current lineage tracing techniques. As correctly pointed out in a recent review [32], these technical shortcomings do not permit the possibility of mesothelial cell transition in vivo to be ruled out entirely. Moreover, mesothelial cells may still contribute to peritoneal fibrosis through their capacity for producing collagen [58]. They can also act indirectly by affecting peritoneal fibroblasts in a paracrine manner. In this respect, it has been demonstrated that lysophosphatidic acid signaling through LPA_1_, a G protein-coupled receptor, stimulates mesothelial cells to produce connective tissue growth factor (CTGF) that subsequently drives peritoneal fibrosis by inducing peritoneal fibroblast proliferation and collagen synthesis [59].

## 6. Fibroblast Involvement in Peritoneal Inflammation

Persistent tissue irritation, inflammation, and fibroblast activation are key features of fibrosis [60]. In this setting fibroblasts not only are effector cells but also contribute signals that control the function of other cell types. Peritonitis is the commonest PD-associated insult to the peritoneum, which is characterized by massive leukocyte infiltration. It is now recognized that the sequence at which different leukocyte subsets arrive in the peritoneum is controlled by a complex intraperitoneal network of chemokines [31]. These chemokines are thought to be produced primarily by mesothelial cells. However, peritoneal fibroblasts are also capable of synthesizing some chemokines and can be as potent in this respect as the mesothelium [61–63]. This activity of peritoneal fibroblasts can help recruit leukocytes during those episodes of severe peritonitis that are associated with extensive mesothelial cell damage and exfoliation [64, 65]. We have demonstrated that HPFB release chemokines MCP-1/CCL2 and IL-8/CXCL8 either constitutively or after stimulation with IL-1*β* and TNF*α*. The response to these macrophage-derived proinflammatory cytokines is mediated through transcription factors of the NF-*κ*B family [61]. The production of neutrophil-targeting cytokines by HPFB is triggered predominantly by IL-1*β* [62]. We have demonstrated that it stimulates the secretion of CXCL1 and CXCL8, classic chemokines for neutrophils, but also of granulocyte colony-stimulating factor (G-CSF) that mobilizes neutrophils from the bone marrow and promotes their survival. On the other hand, we have demonstrated that HPFB can also produce CCL5, a strong chemoattractant for mononuclear leukocytes [63]. The process is critically controlled by IFN-*γ*, which does not stimulate CCL5 itself but synergistically amplifies the effect of TNF*α*. Moreover, by inducing CD40 expression it allows HPFB to synthesize CCL5 in response to CD40 ligand (CD40L) present primarily on T cells.

HPFB can also generate chemokines in response to high glucose exposure. It has recently been observed that the incubation of HPFB with glucose led to a dose-dependent increase in CCL2 mRNA expression [66]. It was preceded by a short-lived increase in the expression of the osmosensitive transcription factor, nuclear factor of activated T cells 5 (NFAT5). However, it is uncertain whether this glucose-induced increase in CCL2 mRNA is mediated by NFAT5. The analysis of peritoneal biopsies revealed no difference in the expression of NFAT5 but still increased expression of CCL2 in the peritoneum of patients undergoing PD compared to those with uraemia but not requiring dialysis.

Taken together, all these observations are in line with the concept of resident tissue fibroblasts acting as sentinel cells that control inflammatory response to tissue injury or infection [67]. In this respect, HPFB can add significantly to transperitoneal chemotactic gradients during peritonitis.

## 7. Conclusions

**Figure 2:**
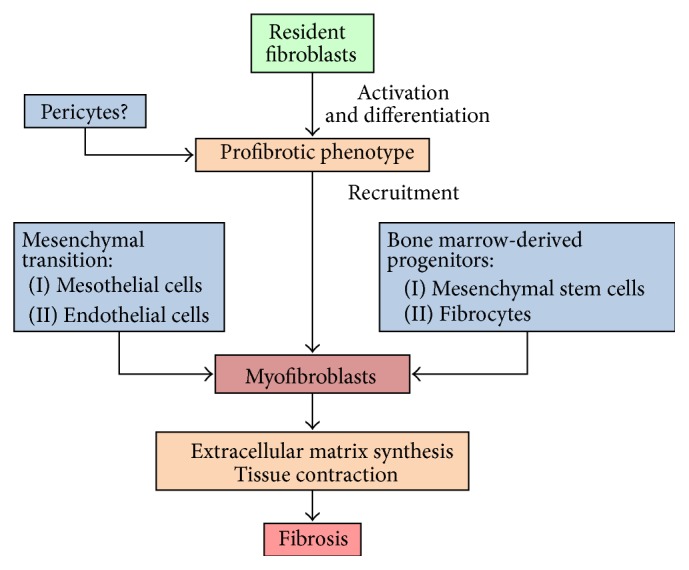
Potential myofibroblast precursors contributing to peritoneal fibrosis in PD. In response to peritoneal irritation or injury, myofibroblasts accumulate in the peritoneum as a result of (i) resident peritoneal fibroblasts activation and proliferation; (ii) transformation of local pericytes; (iii) proliferation and infiltration by resident and circulating fibrocytes; (iv) differentiation of local mesenchymal stem cells; (v) dedifferentiation and mesenchymal transition of peritoneal mesothelial cells; and (vi) dedifferentiation and mesenchymal transition of endothelial cells (adapted from [32, 48]).

Increasing data suggests that collagen-producing myofibroblasts that accumulate in the peritoneum in the course of PD are derived from various precursors (Figure 2). The exact contribution of these cellular sources remains to be established. Of those, resident peritoneal fibroblasts still need to be considered as important predecessors of myofibroblasts. However, given unique immune environment of the peritoneal cavity, site-specific variation in fibroblast transcriptional profiles, and internal heterogeneity, it is essential to better understand the basics of peritoneal fibroblast biology. This is the prerequisite for interventional therapies targeting PD-associated peritoneal fibrosis.
